# Metformin Suppresses Glioblastoma Tumor Growth and Progression Through the AMPK/FoxO3a/Survivin Axis

**DOI:** 10.3390/cells15030310

**Published:** 2026-02-06

**Authors:** Fabiola Cavaliere, Michele Pellegrino, Alessandro Cormace, Sofia Spadafora, Mariarosa Fava, Seung Ho Yang, Jung Eun Lee, Marta Claudia Nocito, Rosa Sirianni, Ivan Casaburi, Cecilia Garofalo, Diego Sisci, Catia Morelli, Marilena Lanzino

**Affiliations:** 1 Department of Pharmacy, Health and Nutritional Sciences, University of Calabria, 87036 Rende, Italymarilena.lanzino@unical.it (M.L.); 2 Department of Neurosurgery, St. Vincent’s Hospital, College of Medicine, The Catholic University of Korea, 93 Jungbudaero, Paldal-gu, Suwon 16247, Republic of Korea; 3 IRCCS Istituto delle Scienze Neurologiche di Bologna, Via Altura 3, 40139 Bologna, Italy

**Keywords:** glioblastoma, metformin, survivin, FoxO3a, AMPK

## Abstract

Glioblastoma (GB) is one of the most aggressive malignant brain tumors. Due to the high invasiveness of this cancer, surgical removal is often not possible, and relapses after surgery are very common, making current treatments ineffective. Developing new therapies or treatment combinations remains a major challenge in managing GB. Metformin (MET), an anti-diabetic medication, has recently gained attention for its potential anticancer effects. To better understand how MET inhibits GB growth at the molecular level, we studied its impact on survivin, a member of the inhibitor of apoptosis (IAP) family that is essential for GB cell survival, resistance to radio- and chemotherapy, and tumor recurrence. Using T98G and U87-MG cell lines, we performed cell viability, migration, and invasion assays, along with Western blot analysis, ChIP assays, and gene silencing experiments to examine key signaling pathways. We found that MET effectively inhibits the growth, viability, and invasiveness of GB cell lines through a molecular mechanism involving activation of the AMPK/FoxO3a/survivin pathway. In vivo studies support these findings, showing increased FoxO3a and decreased survivin in brain tissue sections from metformin-treated mice compared with untreated controls. These results suggest new possibilities for repurposing MET as an adjuvant treatment for GB.

## 1. Introduction

Glioblastoma (GB), a grade IV IDH-wildtype diffuse and astrocytic glioma in adults according to the latest CNS5 WHO classification [1] (formerly glioblastoma multiforme, GBM), is the most common primary malignant brain tumor arising from the neuroepithelial tissue of the brain and glial cells [2]. Currently, given its low incidence (3.20–4.64/100,000 inhabitants), it is considered a rare tumor with a poor prognosis. The late onset of clinical symptoms often delays the diagnosis of GB, and the average survival time is about 15 months from diagnosis, with a 5-year survival rate below 5% [3]. Indeed, GB is characterized by a particularly invasive and infiltrating nature, as well as a high rate of cellular growth, which is poorly counteracted by current therapies. This leads to a significant incidence of post-operative recurrences and an unfavorable prognosis. The current standard treatment involves a multidisciplinary approach, the so-called Stupp protocol, which includes surgical resection of the tumor mass, followed by concomitant and adjuvant radiation therapy and chemotherapy (essentially Temozolomide, TMZ) to delay postoperative relapses [4]. Unfortunately, GB develops innate or acquired resistance to radio- and chemotherapy [5], and unsatisfactory efficacy has also been reported for the newest experimental treatments based on chimeric antigen receptor T cells (CAR-T) or on oncolytic viruses (e.g., poliovirus and Herpes Simplex virus) [6]. Failure of the available therapeutic approaches has been attributed to genetic modifications, metabolic reprogramming, its heterogeneous nature, and the immunosuppressive microenvironment of GB [7,8] as well as to the low tumor accessibility due to both tumor position and to the presence of the blood–brain barrier [9], which all contribute to resistance [5].

Unlike other tumor types where over the past 50 years the prognosis and the patients’ quality of life have been indisputably improved thanks to the identification of the cellular and molecular mechanisms governing their origin and progression, in GB, no significant advancements have been registered since the Stupp protocol became the standard of care in 2005 [10].

Over the past few years, significant effort has been made to identify therapeutic options for GB management. Among the available options, repositioning or repurposing inexpensive “old” drugs already approved for treating other conditions has always attracted considerable attention since discovering and approving new drugs is typically a costly and time-consuming process.

One of the most promising “old” candidates is metformin (MET), a well-known hypoglycemic drug used in the treatment of type 2 diabetes, which has a high safety profile [11]. Since a 2005 retrospective observational study showed that diabetic patients treated with MET had a reduced risk of cancer, the drug has been extensively studied for its potential antineoplastic activity [12]. Although the mechanisms behind its therapeutic effects are not fully understood, it is accepted that MET may inhibit tumor growth and progression by reducing glucose availability and cancer cell dependence on aerobic glycolysis, with an anti-proliferative effect mainly mediated by the inhibition of mitochondrial OXPHOS through AMPK-dependent and -independent mechanisms [13].

Recent evidence indicates that MET may also be a promising anticancer agent in GB. Indeed, MET has been shown not only to cross the blood–brain barrier (BBB) and be bioavailable in the brain [14,15,16,17], but it can also suppress the growth of glioma cells via the AMPK/mTOR signaling pathway, inducing apoptosis [13]. MET’s anticancer effects involve AMPK activation through multiple mechanisms (reviewed in [18]), including the inhibition of mitochondrial complex 1, activation of liver kinase B1 (LKB1), and the induction of reactive oxygen species (ROS) production, which suggests that cellular bioenergetics could represent a potential therapeutic target in GB.

In addition, MET has been reported to interfere with the PI3K/AKT axis, disrupting GB cell migration and invasion [19] and repressing the self-renewal, survival, and proliferation of glioma stem cells [20].

Finally, the treatment of different human GB cell lines with a combination of MET and irradiation, or the chemotherapeutic agent TMZ can inhibit cell proliferation and viability, as well as increase the sensitivity of several GB cell lines to standard radio- and chemotherapy, respectively [13].

Despite several mechanisms behind MET’s antitumor activity in GB having been reported, translating MET use into a clinical setting remains challenging. Therefore, we reveal additional mechanisms through which MET exerts its antitumoral effects in GB, aiming to identify new potential “druggable” markers to target alongside MET, ultimately enhancing the overall standard of care for GB patients.

## 2. Materials and Methods

### 2.1. Cell Cultures and Treatments

Human GB cell lines, U87-MG and T98G, purchased from the American Type Culture Collection (ATCC^®^ CRL-1690™, Manassas, VA, USA) were cultured in MEM media (Gibco™, Thermo Fisher Scientific, Monza, Italy) supplemented with 10% fetal bovine serum (FBS), 1% penicillin–streptomycin, 1% L-glutamine, 1% sodium pyruvate and 1% MEM non-essential amino acids (all from SigmaAldrich, Merck, Milan, Italy) at 37 °C in a humidified 5% CO_2_ atmosphere. Before each experiment, cell cultures were synchronized in phenol red-free (PRF) media containing 3% FBS (PRF-SFM) overnight, then switched to 5% PRF-SFM and treated with 5 or 10 mM MET.

### 2.2. Cell Viability Assay

GB cells were seeded into 96-well plates (3 × 10^3^ cells/well) in growing medium (GM) and, after 24 h, were starved overnight. Cell viability was determined after exposure to increasing concentrations of MET for up to 72 h by adding Thiazolyl Blue Tetrazolium Bromide and subsequently quantifying its metabolite, 3-(4,5-dimethylthiazol-2-yl)-2,5-diphenyltetrazolium (MTT) (Sigma-Aldrich, Merck, Milan, Italy), by measuring the absorbance at wavelengths of 570 nm and 630 nm.

### 2.3. IC_50_

Based on the results of cell viability experiments, the IC_50_ values were calculated using GraphPad^®^ Prism 8 (Version 8.0, GraphPad Software, Inc., La Jolla, San Diego, CA, USA). The inhibitor concentrations were log-transformed, normalized, and expressed as the mean ± SEM within 95% confidence intervals. The R2 square value indicates the goodness of fit.

### 2.4. Trypan Blue Exclusion Assay

U87-MG (3 × 10^4^ cells/well) and T98G (10^4^ cells/well) cells were seeded into 12-multiwell plates in GM. After 24 h, cells were synchronized overnight, then treated with or without MET for 24, 48, and 72 h. Cells were harvested using trypsin and counted using a Countess^®^ II Automated Cell Counter (Life Technologies, Thermo Fisher Scientific, Monza, Italy). Trypan blue exclusion assay was used to determine the number of living and dead cells.

### 2.5. Anchorage-Dependent Growth Assay

U87-MG (2 × 10^4^ cells/well) and T98G (10^4^ cells/well) were plated in duplicate into 6-multiwell plates in 5% PRF-SFM media and treated with or without MET after 24 h. The medium and treatment were renewed every 72 h. After 8 days, the cell colonies were fixed and stained with a Coomassie Brilliant Blue solution, and their density was quantified using a spectrophotometer at a wavelength of 595 nm.

### 2.6. Soft Agar Anchorage-Independent Growth Assay

Cells (5 × 10^3^) were suspended in 5% PRF-SFM media containing 0.35% agarose and seeded in triplicate in 12-well plates with a layer of 2% agarose on the bottom. After 24 h, the cells were treated with or without MET. The treatment was renewed every 48 h. After 14 days, the cell colonies were incubated at 37 °C for 4 h with MTT (400 μL/well) and counted.

### 2.7. RNA Extraction, Reverse Transcription, and Real-Time PCR

Total RNA was extracted using TRIZOL reagent (Life Technologies, Thermo Fisher Scientific, Monza, Italy) and precipitated with isopropanol. Subsequently, 2 µg of each sample were reverse transcribed using the cDNA Reverse Transcription Kit (Life Technologies, Thermo Fisher Scientific, Monza, Italy). Gene expression was measured through real-time RT-PCR using iTaq Universal SYBR^®^ Green PCR Supermix (Bio-Rad, Hercules, CA, USA) and the following primers: h-survivin forward (5′-GGACCACCGCATCTCTACAT-3′) and reverse (5′-GCAGTGGATGAAGCCAGCCT-3′) (Sigma-Aldrich, Merck, Milan, Italy). The expression of the target gene was normalized to the 18S rRNA content in the same sample. Relative gene expression levels were evaluated based on the untreated sample, and the results were reported as n-fold differences in gene expression.

### 2.8. Western Blot Analysis

Total protein, cytosolic, and nuclear lysates were extracted from cell cultures treated with or without MET. Equal amounts of protein (30–50 μg) were resolved on SDS-PAGE gels as previously described [21]. The membranes were incubated with the following primary antibodies: anti-AMPK (D63G4; 5832S), p-AMPK (T172) (D4D6D; 50081S), AKT (11E7; 4685S), p-AKT (S473) (D9E; 4060S), FoxO3a (75D8; 2497S), and p-FoxO3a (all from Cell Signaling Technology Europe, ZA Leiden, The Netherlands); Cyclin D1 (A-12; sc-8396), Survivin (D8; sc-17779), β-actin (AC-15; sc-69879), and GAPDH (O411; sc-47724) from Santa Cruz Biotechnology, Inc., Heidelberg, Germany; and Lamin B (#702972) from Invitrogen, Thermo Fisher Scientific, Monza, Italy. The antigen–antibody complexes were detected using IRDye secondary antibodies (LI-COR Biosciences, Lincoln, NE, USA). Western blot images were acquired and analyzed using the Odyssey FC Imaging System (LI-COR Biosciences, Lincoln, NE, USA).

### 2.9. Wound-Healing Scratch Assay

GB cells were cultured in monolayers in 6-well plates, scratched, and treated with MET. After 12 h, cells were fixed and stained with Coomassie Brilliant Blue, and the wound closure was photographed at 4× magnification using an OLYMPUS-BX51 microscope, Evident Corporation, Tokyo, Japan. The rate of wound healing was calculated using ImageJ software (version 1.54g, National Institutes of Health, Bethesda, MD, USA).

### 2.10. Boyden Chamber Transmigration Assay

Cells pre-treated overnight with MET were detached with Versene (Gibco™, Thermo Fisher Scientific, Monza, Italy) and suspended (U87-MG: 5 × 10^4^ cells/insert; T98G: 10^5^ cells/insert) in the upper compartment of Boyden chambers (8 μm membranes, Corning, Corning, NY, USA) containing SFM, while the wells were filled with 500 μL of GM. After 12 h, the cells that had migrated were fixed with 4% paraformaldehyde and stained with 4′,6-diamidin-2-phenylindole (DAPI, Sigma-Aldrich, Merck, Milan, Italy). The chambers were photographed at 20× magnification using an Optika Pro View digital camera C-D6CC (OPTIKA S.r.l., Ponteranica (BG), Italy) software (version x64, 4.11.20351.20220226), and the cells were counted using ImageJ software (version 1.54g, National Institutes of Health, Bethesda, MD, USA; https://imagej.net/ij/).

### 2.11. Matrigel Invasion Assay

For the invasion assay, the inner membrane of Boyden chambers was coated with 100 μL of Matrigel^®^ Matrix Basement Membrane (Corning, Corning, NY, USA) (1:5 in PRF-SFM). GB cells, which were previously treated for 24 h, were detached and suspended (10^5^ cells/insert) on the upper surface of the Matrigel layer. After 24 h, the invading cells were fixed, stained with DAPI, and photographed at 20× magnification. The cells were then counted using ImageJ software (version 1.54g, National Institutes of Health, Bethesda, MD, USA).

### 2.12. Phalloidin Staining

Cells were seeded on a coverslip in a 6-well plate with GM. After 24 h, they were starved overnight and then treated with or without MET. Following treatment, the cells were fixed with 4% paraformaldehyde, permeabilized with 0.1% Triton X-100, and blocked with 1% Bovine Serum Albumin (VWR™ International, Milan, Italy) in PBS for 30 min. The filamentous F-actin in the samples was stained with Alexa Fluor™ 568 Phalloidin dye (Invitrogen, Thermo Fisher Scientific, Monza, Italy) for 20 min, and nuclei were stained with DAPI. Images were acquired at 40× magnification using an Optika^®^ Pro View digital camera C-D6CC (OPTIKA S.r.l., Ponteranica (BG), Italy) software (version x64, 4.11.20351.20220226).

### 2.13. Gelatin Zymography Assay

The gelatinolytic activity of matrix metalloproteinase (MMP)2 and 9 and their levels in conditioned media were analyzed by gelatin zymography. GB cells were treated as indicated for 24 h, and the conditioned medium was collected and centrifuged to remove cellular debris. A 15 µL volume of each sample was separated on SDS–polyacrylamide gels containing 0.1% gelatin. The gel was washed with washing buffer (50 mM Tris base (pH 7.5), 10 mM CaCl_2_, and 2.5% Triton X-100), incubated overnight in incubation solution (50 mM Tris base (pH 7.5), 10 mM CaCl_2_, and 1% Triton X-100) at 37 °C, and stained with Coomassie Brilliant Blue. The gelatinolytic activity of the MMPs was assessed by measuring the degradation zones, which appear as clear bands against the dark gel background.

### 2.14. Chromatin Immunoprecipitation Assay

GB cells grown in sub-confluent cultures (70%) were shifted to 3% PRF-SFM overnight and then treated with 10 mM MET or left untreated for 12 and 24 h. Nuclear chromatin was cross-linked with 1% formaldehyde for 10 min at 37 °C. The cells were then lysed, and sonicated on ice for 200 s (10 s ON/10 s OFF after each pulse). The chromatin was immuno-cleared with salmon sperm DNA/Protein A agarose beads (Millipore, Merck, Milan Italy) and then labeled overnight with an anti-FoxO3a polyclonal antibody (cat #720128, Invitrogen, Thermo Fisher Scientific, Milan, Italy). The antibody–chromatin complex was precipitated by adding salmon sperm DNA/Protein A agarose beads and reverse cross-linked. Immunoprecipitated DNA was analyzed by RT-PCR using 2 μL of the diluted (1:5) template DNA. When examined in chromatin immunoprecipitation studies, FoxO1 and FoxO3a are physically associated with a segment of the survivin promoter (−1428 nt) that contains the putative binding sites for FoxO [22]. This putative responsive sequence in the human survivin promoter (−1428 nt) was amplified using specific primers: 5′-TGAGCTGAGATCATGCCACT-3′ (forward) and 5′-CTGGTGCCTCCACTGTCTTT-3′ (reverse). The FoxO3a no-binding site (−2269 nt) was amplified using the primers 5′-TTGTTCCTTTCCTCCCTCCTGAG-3′ (forward) and 5′-GTCAACTGGATTTGATAACTGCA-3′ (reverse). All primers were obtained from Merck, Milan, Italy. Real-time PCR data were normalized to those of unprocessed lysates (input DNA). The results were expressed as n-fold differences relative to the inputs.

### 2.15. Immunostaining

A GB orthotopic mouse model, which was previously established by our co-author Seung Ho Yang and his collaborators, was inoculated with 5 × 10^5^ U87-MG cells and treated with or without MET (2 mg/25 g/day) for 4 weeks [23]. Brain tissues specimens were obtained and used for immunostaining to detect FoxO3a and survivin protein expression in the xenograft tumors.

The 2 mg/25 g/day dosage corresponds to approximately 80 mg/kg/day for mice. Based on standard body surface area-based allometric scaling (Km factors: mouse = 3, human = 37), this dose translates to a human equivalent dose (HED) of approximately 6.5 mg/kg/day or ~390 mg/day for a 60 kg adult [24]. This level is within the clinically relevant range of metformin dosing in humans and is comparable to the lower end of the commonly prescribed starting dose (e.g., 500 mg/day).

Tumors were fixed in 4% PFA for 24 h, and mouse tissues were fixed in formalin to prepare paraffin sections. Paraffin-embedded tissue sections were deparaffinized in xylene and gradually rehydrated in 95%, 90%, and 70% ethanol, followed by PBS washes. After deparaffinization and dehydration, the sections were treated with 3% hydrogen peroxide to block endogenous peroxidase activity. They then underwent antigen retrieval and conventional serum blocking. They were then incubated overnight at 4 °C with specific primary antibodies, anti-FoxO3a (cat# 10849-1-AP, Proteintech, Rosemont, IL, USA) and anti-survivin (cat# sc-17779, Santa Cruz Biotechnology, Inc., Heidelberg, Germany) antibodies, and then with goat anti-mouse or rabbit Alexa Fluor 488 or 546 secondary antibodies (Invitrogen, Thermo Fisher Scientific, Monza, Italy). Nuclei were co-labeled with DAPI (Sigma-Aldrich, Merck, Milan, Italy) and imaged using a Zeiss LSM510 (Oberkochen, Germany).

### 2.16. FoxO3a siRNA-Mediated RNA Interference

To deplete FoxO3a (siF3a) transcripts, a validated stealth RNAi (Oligo ID: VHS41092) was used. Cells were cultured in PRF-GM without antibiotics for 24 h and then transfected in suspension with either siF3a or siScramble (150 pmol/dish) using Lipofectamine 2000 (Invitrogen, Thermo Fisher Scientific, Monza, Italy). A Stealth RNAiTM siRNA (siScramble) that does not target any known genes was used as the negative control. All siRNAs were sourced from Invitrogen (Thermo Fisher Scientific, Monza, Italy). After 8 h, the medium was replaced, and silencing efficiency was evaluated for up to 48 h.

### 2.17. Statistical Analysis

Each data point represents the mean ± SEM of three independent experiments. Data were analyzed for statistical significance (*p* < 0.05) using two-way ANOVA, which was conducted using GraphPad Prism 8 (Version 8.0, GraphPad Software, Inc., San Diego, CA, USA).

## 3. Results

### 3.1. Metformin Reduces Viability and Proliferation in GB Cells

**Figure 1:**
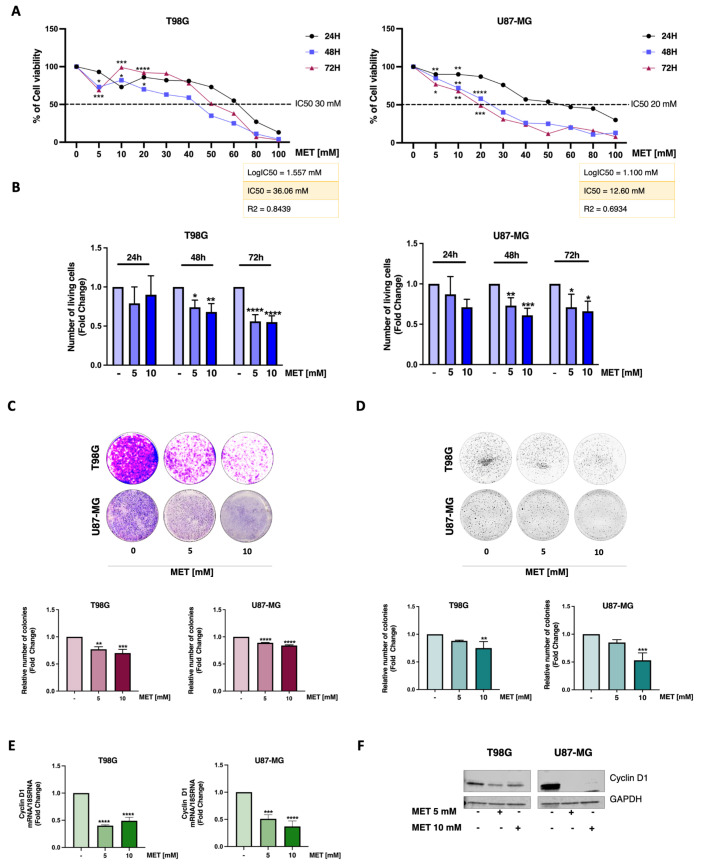
Effects of MET on cell viability and proliferation of GB cells. Human T98G and U87-MG GB cells were treated with or without MET at the indicated concentrations. The IC50 values in the tables were calculated using GraphPad^®^ Prism 8.0 (**A**). Cell viability was assessed using the MTT assay after 24, 48, and 72 h of treatment. (**B**) The proliferation rate was evaluated using the trypan blue exclusion assay and normalized to the control (-). U87-MG and T98G cells were seeded under adhesion (Clonogenic Assay) (**C**) and anchorage-independent conditions (Soft Agar Assay) (**D**) and then treated with MET or left untreated for 8 days (Clonogenic Assay) and 14 days (Soft Agar Assay). Colonies were stained with Coomassie Brilliant Blue solution and MTT, respectively, and counted. Cyclin D1 mRNA levels (**E**) and protein expression (**F**) were assessed by RT-PCR and WB analysis (* *p* < 0.05; ** *p* < 0.01; *** *p* < 0.001; **** *p* < 0.0001).

To determine the optimal MET concentration, the human T98G and the more aggressive U87-MG GB cell lines were exposed to increasing concentrations of MET for up to 72 h. A dose-dependent decrease in cell viability was observed in both cell lines, resulting in comparable and nearly complete cell death with the highest dose (80 mM) used (Figure 1A). Notably, the calculated IC50 values showed that MET had a higher efficacy (i.e., lower IC50) in the more aggressive U87-MG cells (IC50 = 12.60 mM) than in the T98G cells (IC50 = 36.06 mM) (Figure 1A). Trypan blue exclusion assays revealed that even the lowest MET concentrations tested (5 mM and 10 mM) significantly inhibited the viability and proliferation of both cell lines within 48 h (Figure 1B). Consequently, we used these two doses in all subsequent experiments. Colony formation assays further confirmed the anti-proliferative effect of MET under both anchorage-dependent (Figure 1C) and independent (Figure 1D) growth conditions. In both experimental settings, MET significantly reduced the relative number of colonies. These data were further validated by RT-PCR and WB analysis of GB cells treated with MET for 24 h, which showed a significant reduction in both mRNA and protein expression of Cyclin D1, a key regulator of cell cycle progression (Figure 1E,F).

### 3.2. Metformin Decreases the Motility and Invasiveness of GB Cells

**Figure 2:**
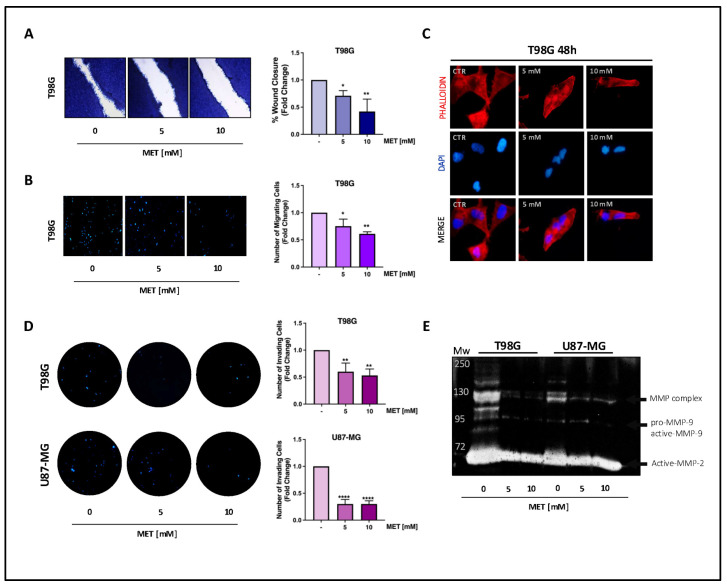
MET inhibits the motility and invasiveness of GB cells. Representative images showing (**A**) closure of a scratch and (**B**) migrated cells after MET treatment for 12 h. The percentage wound closure in each condition was quantified by measuring the area of the same frame at 0 and 12 h after treatment using ImageJ software. (**C**) Immunofluorescence micrograph of fixed T98G cells after treatment with MET for 48 h showing F-actin filaments stained with Alexa Fluor^®^ 488 Phalloidin dye (red), and cell nuclei stained with DAPI (blue). (**D**) Invading cells at the bottom side of the Matrigel layer after 24 h of treatment were fixed and stained with DAPI, and then counted using ImageJ software. The bar graph represents the number of invading cells (mean ± SE) from three independent experiments, each performed in duplicate (**E**). The conditioned media from GB cell cultures treated with MET for 24 h were run on a polyacrylamide gel containing 0.1% gelatin to detect gelatinase (MMP-2 and MMP-9) activity. (* *p* < 0.05; ** *p* < 0.01; **** *p* < 0.0001).

We next tested whether MET may also affect the motility and invasiveness of T98G and U87-MG cells. Wound-healing assays show that MET inhibited wound closure in T98G cells (Figure 2A). Boyden chamber transmigration experiments confirmed that the decrease in wound closure in T98G cells was due to reduced motility, not to a lower proliferation rate (Figure 2B vs. proliferation at 24 h in Figure 1B). These results fit well with the observation that MET treatment induces a significant change in cell morphology. Therefore, Phalloidin staining was used to determine the effects of MET on the polymerization of actin filaments, which are known for their ability to restructure their architecture to enable cell adaptation to the surrounding microenvironment and to guide polarity, morphogenesis, and motility [25]. The treated cells appeared spherical and showed a noticeable loss of cell–cell contacts, along with a reduced thickness and continuity of actin filaments. This indicates that MET inhibits cell motility and changes cell morphology through modulating actin organization in T98G cells (Figure 2C). It should be noted that, although U87-MG cells appeared more sensitive to MET treatment compared to T98G as they required less time to undergo a morphological change (Figure S1A), the wound-healing and transmigration assays did not show any significant reduction in the motility of MET-treated U87-MG cells (Figure S1B,C).

On the other hand, MET significantly reduced the ability of both cell lines to invade an artificial Matrigel^®^ membrane that mimics the extracellular matrix (ECM) (Figure 2D). The effects of MET on cell invasiveness were confirmed by a zymography assay conducted on the conditioned media of both GB cell lines to investigate the secretion and activity of matrix metalloproteases (MMPs). MET treatment decreased the secretion of both the pro- and active forms of MMP-2 and MMP-9 (Figure 2E), providing evidence that MET is a potent inhibitor of GB cell invasive potential.

### 3.3. Metformin Induces FoxO3a Nuclear Translocation in GB Cells

**Figure 3:**
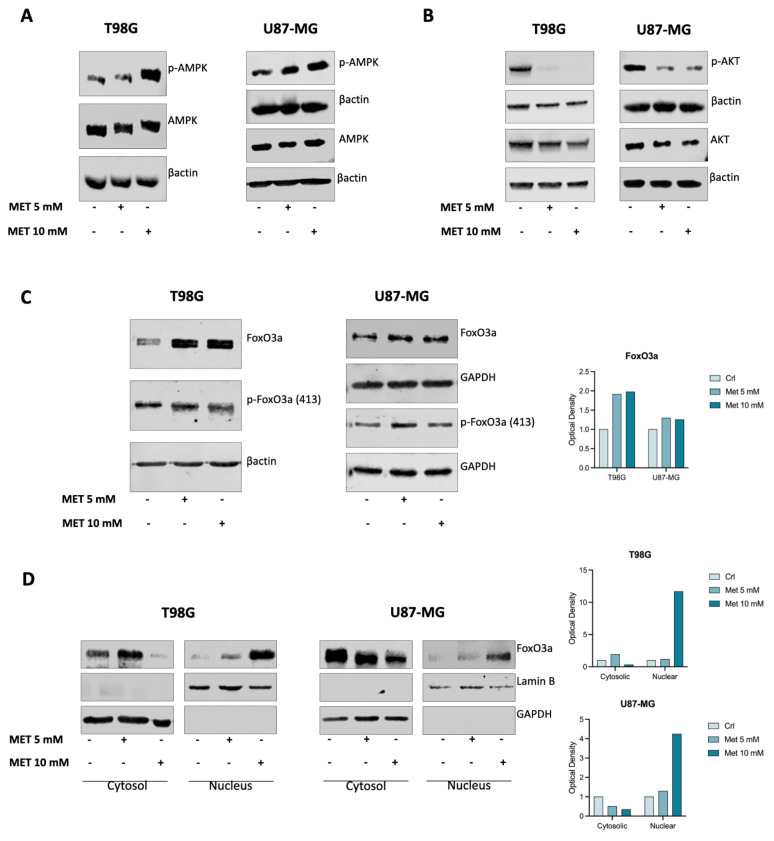
MET triggers the activation of the AMPK pathway, leading to the nuclear translocation of FoxO3a. AMPK phosphorylation and AKT levels in T98G and U87-MG GB cells (**A**,**B**) and the subsequent activation of FoxO3a (**C**) after 24 h of MET treatment were assessed by WB analysis of total lysates. GAPDH was used as the loading control. Nuclear and cytoplasmic lysates were used to determine FoxO3a nuclear translocation (**D**). Lamin B and GAPDH were used as the loading controls for the nuclear and cytoplasmic extracts, respectively. The results are presented as the mean ± SE of three independent experiments.

MET has been reported to inhibit the tumor-initiating potential and self-renewal capacity of patient-derived glioma stem cells by activating FoxO3a via AMP-activated protein kinase (AMPK) [26]. Therefore, we first investigated whether MET could activate the canonical AMPK signaling pathway in our in vitro models. WB analysis of total lysates from cells treated for 24 h with MET demonstrated that, in both GB cell lines used, an increase in AMPK phosphorylation (Figure 3A) was observed, along with a significant inhibition of AKT phosphorylation (Figure 3B).

FoxO3a is a key downstream mediator of the AMPK signaling pathway, and its phosphorylation by both activated AMPK [27] and aberrant PI3K/AKT signaling—which is constitutively active in most cancer cells—leads to FoxO3a inactivation due to its translocation from the nucleus to the cytoplasm. This occurs in several human tumors, including GBs [28], where inactive FoxO3a has been associated with poor clinical outcomes and its expression was inversely correlated with the malignant grade of gliomas [29]. Thus, we evaluated whether MET treatment could activate FoxO3a in our experimental cell models. As expected, MET induced both AMPK activation and inhibition of AKT phosphorylation, leading to induction of FoxO3a protein expression (Figure 3C) due to increased AMPK-dependent phosphorylation of Ser^413^ (Figure 3A), as well as the translocation of FoxO3a from the cytoplasm to the nucleus in both cell lines tested (Figure 3D).

### 3.4. Metformin Reduces Survivin Expression in GB

**Figure 4:**
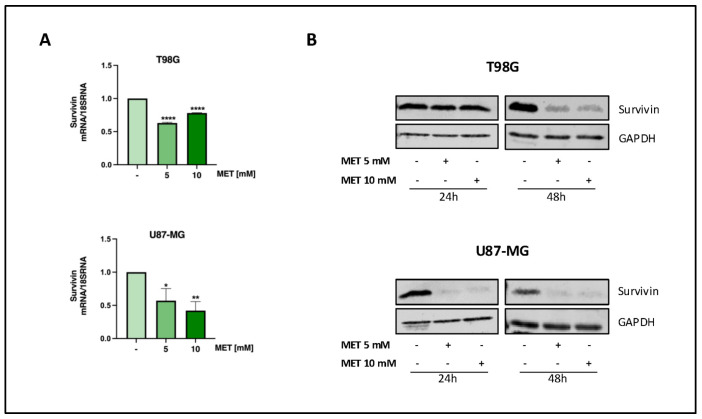
MET decreases survivin mRNA levels and protein expression. (**A**) RT-PCR for survivin mRNA level in GB cells treated with the indicated concentrations of MET for 24 h. Bar graph presents the mean ± SE of three independent experiments (* *p* < 0.05; ** *p* < 0.01; **** *p* < 0.0001). (**B**) WB for survivin protein expression in GB cells treated with MET for 24 and 48 h. GAPDH was used as the protein loading control.

Survivin, a member of the Inhibitor of Apoptosis (IAP) family and also known as baculoviral inhibitor of apoptosis repeat-containing 5 (*BIRC5*), is highly expressed in several tumors, including GB, where it appears to be a negative prognostic marker [30]. Therefore, the effect of MET on the function of this crucial protein was investigated in T98G and U87-MG cells. Our results show that MET significantly reduced survivin mRNA levels in both cell lines as early as 24 h after its administration (Figure 4A), which was accompanied by a substantial reduction in survivin protein expression. Notably, the two cell lines exhibit different response times to the treatment. T98G required 48 h of exposure to MET to show a reduction in survivin protein, while U87-MG demonstrated higher sensitivity, with a decrease in survivin levels already visible after 24 h of MET exposure (Figure 4B).

### 3.5. FoxO3a Inhibits Survivin Gene Transcription in MET-Treated GB Cells

**Figure 5:**
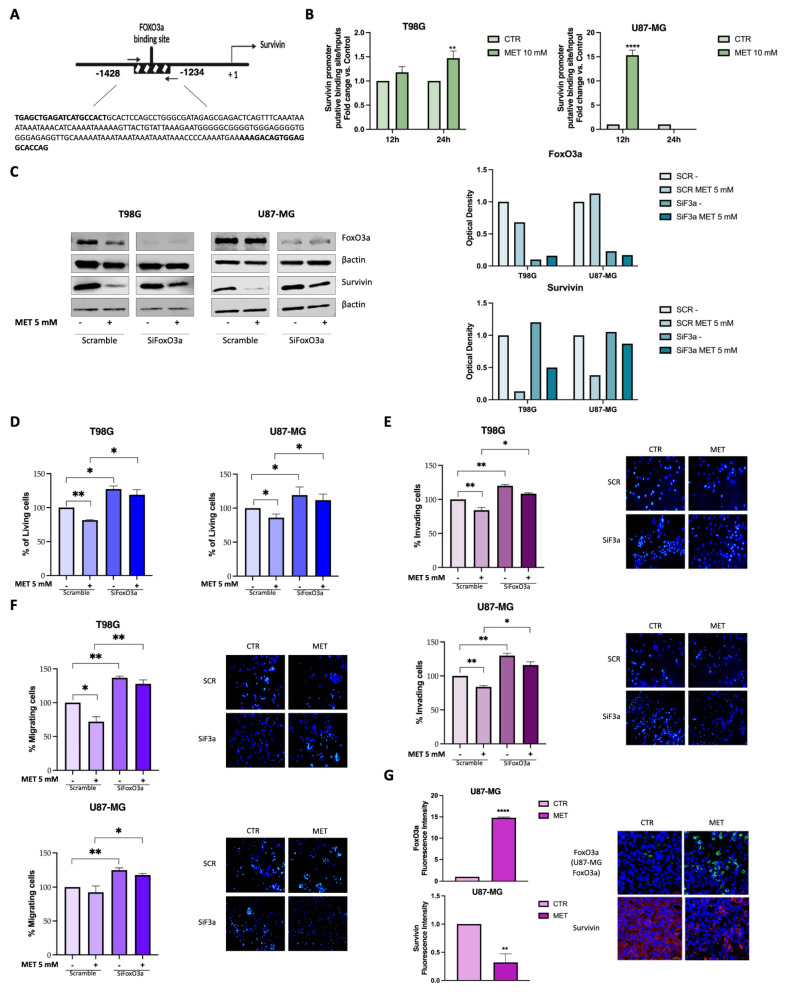
FoxO3a transcriptionally regulates survivin expression by binding to its promoter. (**A**) Survivin promoter bearing a forkhead binding site (FKHE, from nt −1428 to nt −1234). (**B**) ChIP assay for FoxO3a on the survivin promoter. Nuclear extracts from T98G and U87-MG cells treated with 10 mM MET for 12 and 24 h were immunoprecipitated with an anti-FoxO3a antibody, and the immune complexes were amplified by RT-PCR using primers targeting the survivin promoter’s putative binding sites. Bar graph present the mean ± SE of three independent experiments (** *p* < 0.01; **** *p* < 0.0001). (**C**) WB for FoxO3a and survivin expression, (**D**) proliferation, (**E**) invasion, and migration (**F**) in FoxO3a-silenced GB cells after 24 h of MET (5 mM) treatment. Bar graph present the mean ± SE of three independent experiments (*p < 0.1; ** *p* < 0.01; **** *p* < 0.0001). (**G**) Representative images of FoxO3a and survivin immunostaining of tissue sections of U87-MG cell-derived tumors explanted from mice treated with or without MET (2 mg/25 g/day) [23].

Activated FoxO3a has been shown to inhibit survivin transcription by binding to its promoter [22,31]. Therefore, ChIP experiments were performed to determine whether nuclear translocation of FoxO3a induced by MET is followed by FoxO3a recruitment to FoxO-responsive motifs (−1428 nt) on the survivin promoter in GB cells (Figure 5A). Indeed, MET promoted FoxO3a binding to the survivin promoter in both cell lines. Notably, FoxO3a recruitment exhibited different kinetics in the two GB cell lines, somewhat reflecting the relative inhibition of survivin expression observed in Figure 4B in MET-treated cells. Specifically, T98G cells showed a slight, yet significant, increase in survivin promoter occupancy by FoxO3a only at 48 h, while in U87-MG cells, FoxO3a recruitment showed an approximately 15-fold increase as early as 12 h after MET treatment (Figure 5B).

To elucidate the role of FoxO3a in survivin regulation and to clarify the function of the FoxO3a/survivin axis in mediating the inhibitory effects of MET, FoxO3a silencing experiments were conducted in GB cells. The findings indicate that FoxO3a serves as a mediator of MET activity, as its silencing restored the survivin levels that were reduced by MET in both T98G and U87-MG cells (Figure 5C). Furthermore, FoxO3a silencing reversed the MET-induced inhibition of cell viability (Figure 5D) and cell invasiveness (Figure 5E) in both cell lines, as well as cell migration in T98G cells (Figure 5F). According to Figure S1B,C, the inhibition of cell migration in U87-MG cells by MET was not statistically significant, although a reduction trend was observed. Importantly, FoxO3a silencing significantly attenuate this effect (Figure 5F). It should be noted that FoxO3a knockdown alone, independent of MET treatment, also improves cell viability, invasion, and migration. These results suggest that FoxO3a’s involvement in regulating these biological processes in GB cells may depend on additional pathways.

An inverse relationship between FoxO3a and survivin levels was also demonstrated in vivo. As expected, increased FoxO3a levels and decreased survivin protein expression were observed in brain tissue sections from GB-bearing mice treated with or without a clinically relevant dosage of MET (2 mg/25 g/day) (Figure 5G).

## 4. Discussion

GB is the most common and incurable primary brain tumor. Clinical management involves surgery combined with chemotherapy and radiation therapy, but the response to treatments is poor. As a result, drug resistance and tumor relapse occur in nearly 100% of patients, with an average 2-year survival rate of only 6% to 12% [10]. The poor clinical outcomes of current treatments for GB pose a significant challenge for developing new and effective drugs. Over the past few decades, several repurposed drugs—such as antihypertensives, anthelmintics, antibiotics, antihyperlipidemics, anti-inflammatory agents, antimalarials, and antidiabetics—have garnered significant attention in oncology research [32] since their known pharmacological properties and excellent safety profiles make them promising candidates for rapid implementation in tumor treatment.

Among these candidates, the anti-diabetic drug MET has been associated with the restoration of radio- and chemosensitivity in several cancers, including GB.

Yang, S.H. et al. previously showed that MET can partially restore TMZ sensitivity in resistant GB cells by downregulating SOX2; reducing self-renewal, migration, invasion, neurosphere formation, and tumor growth; modifying fatty acid metabolism; and reversing the gene expression changes associated with TMZ resistance [33]. Additionally, they reported that combining TMZ with high-dose metformin improves treatment efficacy by increasing cytotoxicity, inducing apoptosis, and extending survival in mice. This combination may address TMZ resistance by activating AMPK, inhibiting mTOR and AKT phosphorylation, and decreasing FASN levels [23]. Other studies have shown that MET overcomes TMZ resistance by counteracting TMZ-induced AKT–mTOR activation, promoting cell death, and enhancing apoptosis independently of AMPK [34]. Moreover, MET increases the Bax/Bcl-2 ratio, reduces ROS levels, modulates glioma stem cell markers such as CD90, and alters metabolism to induce energy stress [35].

Given that MET is efficiently absorbed into brain tissue, can cross the BBB [15], and exhibits notable neuroprotective properties, we sought to determine whether additional mechanisms contribute to MET’s biological effects in GB.

MET exerts anti-proliferative, pro-apoptotic, and anti-migratory effects in tumor cells, mainly through activation of the AMPK pathway. A widely accepted mechanism of MET is the inhibition of mitochondrial complex I of the electron transport chain, which decreases cellular energy levels (lower ATP-to-AMP ratio). This decrease is detected by AMPK, leading to its activation. Once activated, AMPK triggers several downstream effects, including the downregulation of cyclin D1, a key regulator of the G1-to-S-phase transition, and the induction of the tumor suppressor p53, both of which lead to cell cycle arrest [36]. Another proposed molecular mechanism underlying the anti-tumoral effect of MET involves the inhibition of the phosphatidylinositol 3-kinase/protein kinase B (PI3K/Akt) axis [20,37].

According to Ibrahim R.S. et al. [17], the activation of AMPK combined with PI3K/Akt pathway inhibition in MET-treated GB cells, including the T98G [38] and U87-MG [19] cell lines, has been associated, to varying extents, with a decrease in cell proliferation, survival, migration, invasion, as well as in cell cycle arrest and apoptosis.

The nuclear transcription factor and oncosuppressor FoxO3a, a member of the subfamily of forkhead box transcription factors that mediate different cellular processes, is a downstream target of both AMPK and AKT. MET has been shown to upregulate and/or activate FoxO3a in several cancers, including colon [39], pancreatic [40], breast [41] and cervical cancer [42]. MET has also been shown to suppress the tumor-initiating ability and self-renewal of patient-derived glioma stem cells by activating FoxO3a via AMPK [26]. However, the specific mechanism(s) through which FoxO3a inhibits tumor growth and progression in MET-treated GB cells have not been fully elucidated.

After confirming the antiproliferative and anti-invasive effects of MET on T98G and U87-MG GB cells [13,19], as well as demonstrating that MET induces AMPK-dependent phosphorylation resulting in FoxO3a nuclear translocation (Figure 3) [26], we focused on the FoxO3a target gene survivin as a potential mediator of MET-induced effects in GB.

Survivin, the smallest member of the IAP family, is thought to be involved in the increased proliferation, survival, and metastasis of cancer cells, as well as other key hallmarks of cancer [43]. Growing evidence indicates that survivin expression is highly deregulated in cancer and is linked to the aggressive nature and higher clinical–pathological grade of GB, as well as its radio- and chemoresistance [44,45,46]. Survivin has been defined as a nodal protein due to its crucial role in suppressing apoptosis and controlling mitosis [47], in maintaining stem cells [48], in angiogenesis [49], and in invasion and metastasis [43]. In particular, survivin inactivation by the specific inhibitor YM155 dramatically suppressed the proliferation of U87 GB cells, reduced their anchorage-dependent and -independent growth, and weakened their invasive and metastatic abilities [50].

The regulation of survivin expression is highly complex, with several transcription factors controlling its transcription [51]. Among these, FoxO3a has been reported to repress survivin transcription and protein expression by binding to a putative FoxO-responsive sequence located in the survivin promoter in human neuroblastoma cells [31]. Here, we show that survivin is transcriptionally regulated by FoxO3a in GB cells in response to MET treatment. Silencing experiments confirmed this relationship, as FoxO3a depletion markedly increased survivin expression in MET-treated cells and attenuated MET-mediated inhibition of GB cell proliferation, migration, and invasion (Figure 5).

**Figure 6:**
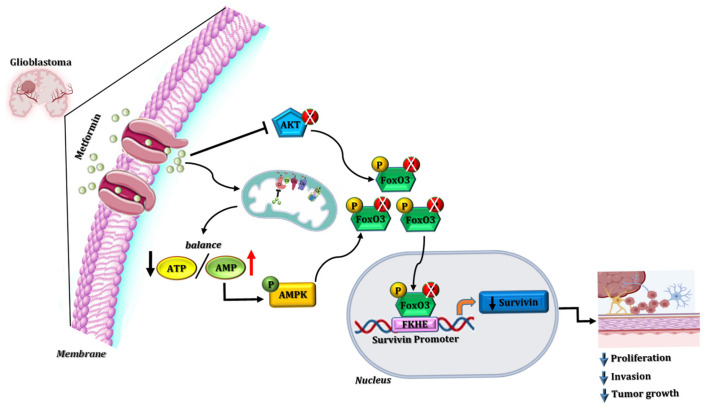
MET inhibits GB growth and progression through the FoxO3a/survivin axis. After uptake by the organic cation transporter (OCT), MET causes a decrease in the ATP/ADP ratio by inhibiting mitochondrial respiration, leading to the activation of AMPK [13], which induces FoxO3a phosphorylation and its subsequent nuclear translocation where it binds to a FHRE core sequence located in the human survivin promoter. This decreases its expression, which consequently inhibits tumor growth and progression.

Figure 6 schematically depicts the proposed mechanism through which MET promotes FoxO3a expression and nuclear translocation. Once in the nucleus, FoxO3a binds to FKHE-responsive elements within the survivin gene promoter, leading to repression of survivin transcription and, consequently, reduced GB cell proliferation, migration, and invasion.

Our in vivo analyses supported our in vitro findings, revealing increased FoxO3a levels and reduced survivin expression in MET-treated U87-MG-derived tumors compared with controls. Immunohistochemical evaluation was performed on brain tissue from a glioblastoma orthotopic mouse model that was previously established by Yang, S.H. et al. Using this model, they demonstrated that MET monotherapy significantly prolonged mouse survival (55.2 days vs. 43.6 days in control animals) [23]. These survival benefits are consistent with our observation of increased FoxO3a and decreased survivin levels in GB tumor tissue, supporting a mechanistic link between MET treatment and modulation of pro-survival signaling pathways in vivo.

The potential use of MET in GB aligns with the growing interest in targeting GB metabolism. In fact, it is known that GB undergoes extensive metabolic reprogramming, which facilitates tumor growth and therapeutic resistance by altering glycolysis and nutrient utilization [52,53]. Targeting this vulnerability with MET, which activates AMPK and shifts metabolism toward oxidative phosphorylation [13], represents a promising strategy. There are ongoing clinical trials (IDs: NCT02780024, NCT05929495, and NCT04945148) investigating MET’s therapeutic benefits using various GB treatment protocols. By activating the AMPK/FoxO3a axis and subsequently suppressing survivin expression, MET could enhance GB cell sensitivity to TMZ, positioning survivin as a central node connecting metabolism and therapy resistance. This hypothesis is supported by recent clinical developments that demonstrated that survivin-targeting immunotherapies, such as the SurVaxM vaccine, are showing promising safety and efficacy in combination with TMZ in Phase II trials for newly diagnosed GB (ID: NCT02455557) [54]. Collectively, these findings underscore the potential of targeting metformin–FoxO3a–survivin modulation for metabolic subtype-specific GB therapies.

It is important to note that, while the MET concentrations used in our in vitro experiments are higher than those typically achieved clinically, this approach allowed for a robust assessment of the underlying mechanisms [55]. Importantly, the dosing regimen for the in vivo studies was carefully scaled to reflect clinically relevant human exposures. Nonetheless, differences in pharmacokinetics, administration routes, and tissue distribution across species—especially in the brain—should be considered when interpreting these results. Thus, our findings offer valuable mechanistic insights but should not be viewed as direct evidence of metformin distribution or effects in the human brain [56].

Although activation of the AMPK–FoxO3a pathway by metformin in glioblastoma has been previously reported [26], our study adds to this knowledge by demonstrating that survivin is a direct transcriptional target of FoxO3a in this setting. While this represents an incremental advance, it provides important mechanistic insight into how metformin exerts its anti-tumor effects and refines our understanding of the FoxO3a–survivin axis in GB.

The mechanism proposed for GB cells where MET downregulates survivin expression could also occur in other cancer types. In fact, MET has been reported to reduce survivin protein levels by decreasing its stability and promoting its proteasomal degradation via activation of the AMPK/PKA/GSK-3β-axis in non-small cell lung cancer [57]. Similarly, the AMPK/mTOR pathway has been shown to mediate MET-induced inhibition of survivin in gastric cancer cells [58]. Interestingly, MET appears to reduce survivin mRNA transcripts in ovarian cancer cells [59] and in hepatocellular carcinoma cells when given in combination with epigallocatechin-3-gallate [60]. However, the mechanisms underlying the MET-dependent suppression of survivin mRNA were not investigated in these studies; therefore, it can be hypothesized that MET-mediated inhibition of survivin transcription by FoxO3a is a more general mechanism that occurs across several cancer types rather than in GB alone.

## 5. Conclusions

GB is one of the most challenging cancers to treat due to its aggressive nature and resistance to conventional therapies. Therefore, there is a pressing need to identify additional therapeutic targets to improve GB treatment efficacy.

FoxO3a and survivin play essential roles in controlling apoptosis and cell survival. While FoxO3a serves as a tumor suppressor by promoting apoptosis, survivin inhibits apoptosis and encourages tumor growth. The inverse relationship between FoxO3a and survivin levels seems to be a key factor in the aggressiveness and progression of GB. In this study, we demonstrated that activation of FoxO3a by MET leads to transcriptional repression of survivin expression, which explains the decreased proliferation, migration, and invasion observed in MET-treated GB cells.

Therefore, therapeutic strategies that restore FoxO3a activity or enhance its nuclear localization can downregulate survivin, promoting apoptosis and inhibiting tumor progression. Nevertheless, the simultaneous reactivation of FoxO3a and direct targeting of survivin (e.g., with antisense oligonucleotides, small-molecule inhibitors, or immunotherapy) may yield synergistic effects and offer promising avenues for developing more effective therapies for GB patients by sensitizing the cancer to chemotherapy and radiotherapy and potentially improving the clinical outcomes.

## Data Availability

The original contributions presented in this study are included in the article/Supplementary Materials. Further inquiries can be directed to the corresponding authors.

## Supplementary Materials

The following supporting information can be downloaded at: https://www.mdpi.com/article/10.3390/cells15030310/s1, Figure S1: Effect of MET on U87-MG cells.
