# Supplementary material for: Metformin Suppresses Glioblastoma Tumor Growth and Progression Through the AMPK/FoxO3a/Survivin Axis
Source: Cells. 2026 Feb 6;15(3):310. doi: 10.3390/cells15030310 (PMC12897146; doi:10.3390/cells15030310)
Supplement: Supplementary file 1 [file cells-15-00310-s001.zip › cells-4074152-supplementary.pdf]

Supplementary Materials

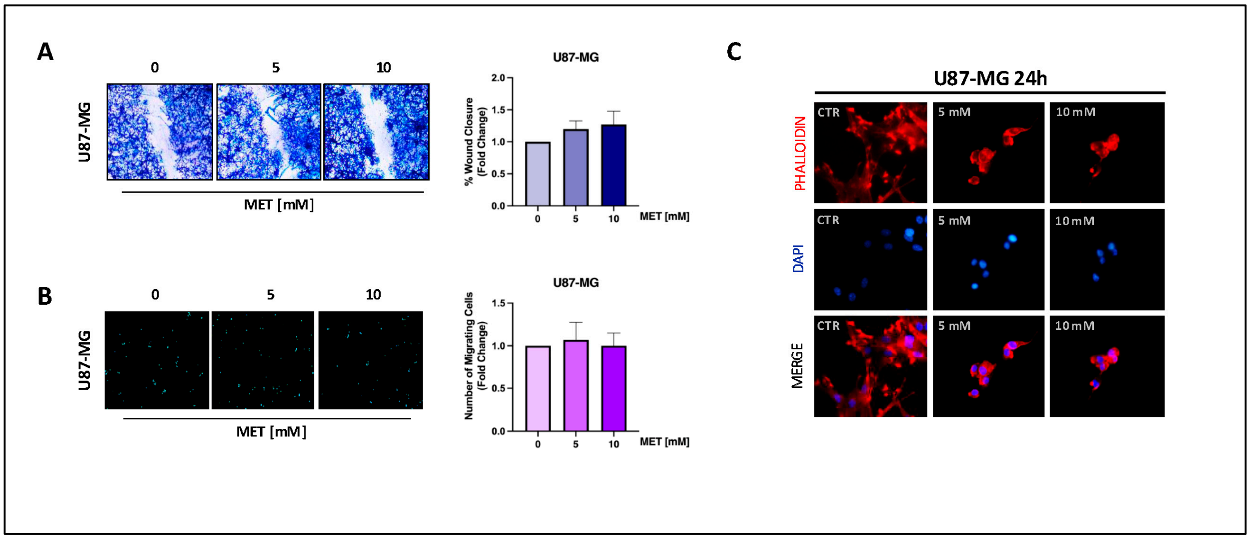

Figure S1. Effect of MET on U87-MG cells. (**A**) Wound closure, and (**B**) migrated U87-MG cells after 12 hours of MET treatment. Wound closure was measured with ImageJ software and shown as a percentage. (**C**) Morphological changes after 24 hours of MET exposure.
